# Storytelling and poetry in the time of coronavirus

**DOI:** 10.1017/ipm.2020.36

**Published:** 2020-05-14

**Authors:** Elizabeth Barrett, Melissa Dickson, Clare Hayes-Brady, Harriet Wheelock

**Affiliations:** 1 Child and Adolescent Psychiatry, Children’s University Hospital Temple St, Dublin 1, Republic of Ireland; 2 UCD Child and Adolescent Psychiatry, University College Dublin, Dublin 4, Republic of Ireland; 3 Department of English Literature, University of Birmingham, Birmingham, UK; 4 School of English, Drama and Film, University College Dublin, Dublin 4, Republic of Ireland; 5 Royal College of Physicians of Ireland, Dublin 2, Republic of Ireland

**Keywords:** Narrative in medicine, medical education, compassionate care, medical humanities, clinician burnout

## Abstract

The coronavirus crisis occurs at a time when many clinicians have already experienced burnout. One in three Irish doctors were suffering from burnout in the 2019 National Study of Wellbeing of Hospital Doctors in Ireland; rates are also high in Irish Psychiatry. We present a perspective on the use of narrative in medicine and recognise that storytelling, and the patient history are very much at the heart of medicine. Clinician storytelling, such as Schwartz Rounds and Balint group work, has very much come to the fore in Irish Psychiatry and in training. Projects such as MindReading have explored overlaps between clinicians, humanities experts and experts by experience. We give an overview of some approaches from the movement around narrative in medicine to bolster this. We explore why clinicians write as ways to support identification, catharsis and a way to process experiences. Clinicians and patients may also use literature and poetry to promote coping. The historical context and practical strategies are highlighted, particularly with reference to poetry use during the current crisis.

## Introduction: changing contexts

and anyway it’s just the same old story -

a few people just trying,

one way or another,

to survive.

Mostly, I want to be kind.

And nobody, of course, is kind,

or mean,

for a simple reason.

And nobody gets out of it, having to

swim through the fires to stay in

this world.

― Mary Oliver, *Dream Work*

The current coronavirus crisis occurs at a time when many clinicians have already experienced burnout. One in three Irish doctors were suffering from burnout in the 2019 National Study of Wellbeing of Hospital Doctors in Ireland (Hayes *et al*. 2019), while rates are also high in Irish Psychiatry (McNicholas *et al*. 2020). As a profession, there is a need to explore new frameworks of systemic support. This is perhaps an opportune time to reflect on our current structures and consider methods of protecting and sustaining the mental as well as the physical well-being of healthcare workers. Many of us are confronting the vulnerabilities not only of our political, economic and public health systems, but also of our own bodies and minds, and those of our loved ones. At the same time that we face a tragic loss of life, often in extremely difficult circumstances, we are struggling with how that loss is to be grieved, remembered or even counted. Beyond the daily statistics and factual briefings, an alternative, qualitative medium is necessary to express and explore the spectrum of unique, often intensely personal experiences of this pandemic, and to provide both solace and outlet to those afflicted.

Charon in 2012 writes that ‘recognizing clinical medicine as a narrative undertaking fortified by learnable skills in understanding stories has helped doctors and teachers to face otherwise vexing problems in medical practice and education in the areas of professionalism, medical interviewing, reflective practice, patient-centred care, and self-awareness’ (Charon 2012). Many approaches to mental health have embraced the practice of narrative medicine. Schwartz Rounds, for example, are about sharing the human stories at the heart of medicine and are evidence-based approaches shown to underscore the well-being of clinicians (and thus, by extension, of their patients). These are evidenced, structured interventions shown to support staff and impact on compassionate care in hospitals (Point of Care Foundation; Maben *et al*. 2018; Silke, 2019). Balint groups involve a number of clinicians coming together to share a single story about a doctor–patient interaction and collectively reflecting on it. Balint and reflective practice groups are one way of offering structured, regular, predictable peer support (Salinsky, 2009). Other groups have recognised the importance of clinicians recording their experiences, whether in the form of autobiography or as fictionalised representations of their experiences (Faust, 2019). The JAMA Network, in 2019, produced a short documentary, The Making of The House of God, detailing the book’s origins and the people and events that inspired its stories (JAMA Network, 2019). Very evident in this interview is an understanding of why clinicians share stories and why clinicians write as ways to support identification, as a means of catharsis and processing experiences.

The potential impact of narrative on clinicians goes beyond the reflective practices and invited empathy of memoir; fiction and poetry, too, have their uses in returning to us the fundamental strangeness of the human condition. Indeed, as Oyebode points out, the aim of the fiction writer writing about madness is ‘to distance the reader from the symbol of madness’ (Oyebode, 2009). For clinicians, this distancing invites space to recognise the shock of deviation from the norm as experienced in burnout. It is, as Oyebode notes, an ‘opportunity to stand back from [the] world, to contemplate it, before once again immersing ourselves in it, for better or worse’ (Oyebode, 2009). In this case, fiction’s role is not to explain, or to comfort, but to accommodate difficulty, and give breathing space to contradiction.

## Practical strategies in utilising poetry and storytelling

Perhaps now is a good time to consider utilising such strategies for both clinicians and patients. Many authors claim that within medicine, humanities and poetry are often seen as exclusively extracurricular pursuits (Davies, 2018). Davies states that ‘within palliative care, however, there has been a long-standing interest in how poetry may help patients and health professionals find meaning, solace and enjoyment’. Poetry has been reported to improve listening, attentiveness, observation and analytical skills. It allows early career doctors to reflect on the patient experience of illness. It can foster creativity, empathy and the realisation that much is outside the gift of clinicians (Shapiro, 2004; Stuckey, 2010).

The MindReading project is a collaborative, interdisciplinary educational effort that was founded in 2017. The primary aim is to explore productive interactions between literature and health both historically and in the present day through international academic collaborations. There is a strong focus on the overlaps between physical and mental health and the impact of illness on sense of self. Its annual conference involves patients, poets, interprofessional clinicians from a range of medical and mental health disciplines, literary scholars, historians, medical humanities scholars, creative writers, and experts by experience. It is truly an example of interprofessional education (IPE), which aims to improve patient care through an interactive learning process: ‘IPE occurs when two or more professions learn with, from and about each other to improve collaboration and the quality of care’ (CAIPE, 2002).

To date, MindReading’s main activities have functioned as independent events brought together by their intent to explore the best ways of drawing on the insights of historical and literary research in contemporary medical practice in the field of physical health and mental health. Participants have shared their research and experiences, and an online resource collection of works nominated by participants has been assembled (available at http://www.ucd.ie/medicine/capsych/mindreading/). There are also ongoing collaborations across groups and with expert-by-experience groups in several universities.

In past meetings, MindReading has explored aspects of health relevant to the current pandemic, from the experience of illness in clinicians, to perinatal mental health, and even burnout from clinical, philosophical and historical perspectives. The 2019 meeting at St Anne’s College, Oxford focused on mental health and adolescence, and ways in which poetry and storytelling might mitigate and mediate youthful experiences of trauma (Diseases of Modern Life, 2019). This year’s (now postponed) meeting was to take place in April at the Royal College of Physicians of Ireland (RCPI) in Dublin and the Lexicon Library, continuing the focus on adolescent health and narrative and exploring the history of vaccination and science denialism.

It is a central premise of MindReading that both literature and clinical medicine deal with issues surrounding subjective identity, selfhood, and social and cultural determinants of health and well-being. This is particularly brought to the fore in the complex relationships between medical and mental illness, the patient, and the physician. At times, this may involve engagement with questions of pain, trauma and the disruption of selves through the use of language, narrative structures and creative expressions. As well as providing insight into these most basic and universal of human concerns, and the attitudes and experiences of people coping with illness or making decisions about their health, MindReading asks how literature might usefully inform the science and practice of clinical medicine. Lived experience and service user experts by experience groups have been central to the project. The current crisis highlights the need for such interdisciplinary approaches and the impact of a physical health crisis on the mental health of the entire population (MindReading, 2017; UCD, 2017; Barrett, 2019).

Other groups have taken similar approaches to utilising humanities approaches in clinical settings. For example, Dr Sophie Ratcliffe at Oxford University has published on the ongoing *The Poetry of Medicine* project (Ratcliffe, 2016). This involves a series of one-day workshops ‘providing a space in which those caring for others could consider the challenges and pleasures of their working life’. Participants rated the course highly, though the author noted the practical challenges in terms of continuing professional development and approval of these events, stating that the ‘sort of discussion space is critically important to the well-being of those who work in the health service. Our challenge is to find a way of highlighting the importance of this kind of non-target led, reflective, supportive space in an environment that seems, all too often, to be driven by the requirements of organisation….’ (Ratcliffe, 2016).

## Poetry during the pandemic

Each day since 30 March 2020, the RCPI Heritage Centre in Dublin, in collaboration with the MindReading project, has published a recording of a poem to their Twitter feed with the hashtag #PauseforaPoem. Its intent is to enable readers to find some relief from the new daily realities with which we are struggling, by momentarily focusing the attention elsewhere, and engaging the mind with other, more calming, ideas and structures. The notion of the ‘pause’ here, particularly as it appears amidst an ever-updating Twitter feed of information, emphasises this act as one which seeks to move the reader into different temporal structures and patterns, as poetry serves as an avenue for diversion, distraction, and the pleasurable immersion of the reader in the stories and spaces of other places and times.

Why poetry specifically? Readers have, of course, long sought the comfort and solace of poetry in times of stress and grief. The philosopher and political economist John Stuart Mill (1806–1873), for example, declared that the poetry of William Wordsworth was ‘a medicine for my state of mind’, from which he drew ‘a source of inward joy, of sympathetic and imaginative pleasure’ as he struggled with what he termed ‘a crisis in my mental history’ (Mill, 1847). Queen Victoria’s *album consolativum*, compiled after the death of Prince Albert, included extracts from Alfred Lord Tennyson’s prose poem *In Memoriam*, itself written to express his grief over the loss of his friend Arthur Hallam, which she claimed offered her solace in her own grieving state (Purton & Page, 2010). Reading, and more particularly poetry, represents here the stay of activity and the punctuation of the industrialised time of an intensive working day with alternative temporal structures, rhythms and imaginative fields of play. Such an episodic psychological movement between worlds is refreshing, relieving and sustaining. It is also potentially transformative, as it increasingly interrupts, and gives alternative meanings to, reality.

Poetry pays attention to experiences of the body and sensations within the mind in ways that echo medical practice. In the 20th century, William Carlos Williams, a poet and community physician, deployed a clearly medical gaze in his poetic attention to the (particularly female) body and the world (Schnur, 2016). Williams’ ‘On the Road to the Contagious Hospital’, from *Spring and All*, written in the aftermath of the 1918 influenza pandemic (Mariani, 1981), turns this clinical gaze on to the natural world’s struggle towards rebirth after a period of hibernation that feels particularly resonant in this period, reminding the reader that our present struggles have a long history. It also reminds us that our own responses as a culture to the outbreak of disease have a longer historical context, in which pain and suffering break through the compartmentalisation of psychiatric, environmental and literary history, and redraw the connections between physiological, psychological and social health. Contemporary readers, too, find meaning in poetry both individually and collectively; Jill Bialosky’s P*oetry Will Save Your Life*, for example, ties critical moments in her life to the transformative verses she read (Bialosky, 2017). The current crisis has prompted a noticeable media and social media turn to the arts, especially poetry, which suggests an emerging awareness of how poetry can return us viscerally to ourselves in strange times. We might think, for instance, of Patrick Stewart’s daily reading of one of Shakespeare’s sonnets on the social media platform Instagram, where the first of these recitations, Sonnet 116, has been viewed over 475,000 times at the time of writing (https://www.instagram.com/p/B-A3NY2haqq/). Elsewhere, President Michael D Higgins shared a 1993 poem of his own writing, ‘Take Care’ on Facebook on 25 March (https://www.facebook.com/PresidentIRL/posts/3464681550225754)). Long-established poets, led by former UK Poet Laureate Carol Ann Duffy, are contributing work to the project Write Where We Are Now (https://www2.mmu.ac.uk/write/) as a living record of the unfolding situation, and the *LA Review of Books* focused a full edition on ‘a small group of critical thinkers, artists, and poets’ who could ‘reveal their own unique tensions, fears, and even doubts about the types of interventions the world needs right now’ (Evans, 2020). Writing in the body of the edition, political theorist Wendy Brown writes ‘As exhausted health workers keep bodies alive, the creatives – musicians, artists, poets, and storytellers – lift and soothe spirits. Science writers translate the discoveries, trajectories, and unknowns; journalists worthy of the name keep rumors and conspiracies at bay, explain new policies, count and name the dead’ (Brown, 2020). Inevitably, given the ramping up of the crisis across Europe and America in April (incidentally also National Poetry Month in the United States), Eliot’s immortal riposte to Chaucer has been ubiquitous, with variations on the phrase ‘April is the cruelest month’ appearing everywhere from the New York Times (Oestrich, 2020) to the Irish Independent (Sexton, 2020). From seeking new meaning in old texts to bending language to new forms, poetry has provided a point of contact for people in isolation and on the frontlines, struggling to find words for a new reality.

## Conclusion

High rates of doctor burnout, even before the advent of the COVID-19 pandemic, should worry all of us. This is especially true given the links between clinician burnout, compassion fatigue and patient care. Interprofessional approaches, and embracing the arts in medicine, may offer avenues of support and hope. Such avenues may take the form of a pause or escape from daily life, a momentary disinvestment of the self in search of relief, or they may provide an imaginative field for experimentation, self-reflection and self-definition. In both cases, narrative provides materials we may draw upon to manage and to evaluate our experiences.
